# Study of prevalence of Human T cell lymphoma virus-I/II in West India blood donors and high risk persons

**DOI:** 10.4103/0973-6247.67025

**Published:** 2010-07

**Authors:** Amit Agrawal, B. H. Parmar, M. D. Gajjar, N. M. Bhatnagar

**Affiliations:** Director Technical, IMA blood bank of Uttarakhand, Dehradun, India; 1 Department of IHBT, BJMC & Civil Hospital, Ahmedabad, India

Sir,

Blood transfusion services have now become the integral part of health care system throughout the world. Safe blood transfusion is the major goal of blood transfusion services and an uninterrupted supply of safe blood in an efficient, coordinated and cost-effective manner to all those who need it is recognized as essential function of health providers.[1,2]

A safe blood supply is a critical component in improving health care and in preventing the spread of infectious disease globally. Millions of lives are saved each year through blood transfusion. The main concern is related to the risk of transfusion transmissible diseases due to unsafe transfusions. This results from blood collection from unsafe donors, the lack of quality system in blood transfusion services, poor laboratory procedures in blood group serology and inadequate testing of donated blood for transfusion transmitted infections (TTI), errors in the administration of blood and a lack of access and appropriate clinical use of blood and blood products for patients requiring transfusion[3]

HTLV-I and II are human retroviruses with worldwide distributions. Like HIV, infection with HTLV-I/II is persistent retroviral infections and is life-long. To avoid HTLV transmission by transfusion, screening of blood units for HTLV-I/II infection has become mandatory in several western countries. Reports on the prevalence of HTLV among the Indians are few.[4]

This study was done with the aim to establish the baseline data in western Indian blood donors through a sero-survey and this could be of immense value in understanding the epidemiology of HTLV-I/II infection in a developing country like India. A preliminary study was also done to see the prevalence of HTLV-I/II infection in high risk population attending Voluntary confidential Counseling and Testing (VCCT) centre. This study could add a new dimension for further research and management establishing co-relation between HIV-I/II and HTLV-I/II infection.

A 6 month prospective study was conducted in The Department of Transfusion Medicine at the tertiary care institute collecting average 18000 units annually from replacement and voluntary blood donors. A total of 267 donors including 131 voluntary blood donors (every 5th donor), attending the outdoor donation blood donation camp and 136 replacement blood donors (every 15th donor) and 267 persons referred to VCCT centre from various Non-Government Organization (NGO), wards and various others were included in the study. At the time of personal interview, they were subjected to a set of questions that includes the information that could be useful while assessing the donors in terms of HTLV-I/II infection, and informed written consent was taken.

All samples were tested for anti HTLV-I/II by ELISA in addition to ELISA for HIV-I/II, HBV, HCV, and peripheral smear for malaria and TPHA for syphilis. Out of the total 534 samples tested, none of the sample was positive for HTLV-I/II [Table 1]. One voluntary donor sample was positive for HBsAg and one replacement donor sample was positive for HIV-I/II. Out of the total 267 VCCT samples tested, none of the sample was positive for HTLV-I/II. 68 samples were found to be positive for HIV-I/II, 9 samples for HBsAg, and 8 samples for HCV. The difference was highly significant in relation to HIV-I/II positivity among various income groups (*P*<0.01) and significant when various education groups were compared (*P*<0.05)

**Table 1:** Distribution of study groups in relation to TTI seropositivity

Study Groups	No. of cases	TTI Seropositivity			
		HIV-I/II	HBV	HCV	HTLV-I/II	
Donors	267	1(0.37%)	1(0.37%)	-	-
Voluntary	131(49.06%)	-	1(0.76%)	-	-
Replacement	136(50.94%)	1(0.74%)	-	-	-
Male	260(97.38%)	1(0.38%)	1(0.38%)	-	-
Female	7(2.62%)	-	-	-	-
VCCT referred persons	267	68(25.47%)	9(3.37%)	8(3.0%)	-
Male	193(72.28%)	45(23.32%)	4(2.07%)	8(4.15%)	-
Female	74(27.72%)	23(31.08%)	5(6.76%)	-	-

In the present study, no cases were found to be HTLV-I/II positive among blood donors and high risk behavior persons. This may be because of low prevalence of HTLV-I/II in India or due to small sample size in the present study. Thus, more studies with larger sample size are required for finding actual prevalence and for implementing anti-HTLV-I/II testing as a routine among blood donors.
